# Association between schizophrenia and syphilis: a retrospective study in Xiamen, China

**DOI:** 10.1186/s12888-018-1869-6

**Published:** 2018-09-03

**Authors:** Qiao Zhang, Jia-Jiang Xie

**Affiliations:** Xiamen Xianyue Hospital, Xiamen Mental Health Center, Xiamen, 361004 Fujian China

**Keywords:** Schizophrenia, Syphilis, Social function, Biochemical values

## Abstract

**Background:**

Previous studies have shown that patients with mental illnesses such as schizophrenia have a higher risk for syphilis infection. However, the clinical characteristics of psychotic patients who are infected with syphilis remain unknown. The aim of this study was to investigate the prevalence of syphilis in psychotic patients in Xiamen and to compare the social function and serum biochemical markers between schizophrenia patients with and without syphilis.

**Methods:**

There were a total of 1586 psychotic patients screened for syphilis from May 2016 to August 2017 in Xiamen Mental Health Center. We retrospectively studied 87 schizophrenia patients in this study. The NOSIE-30 score and serum biochemical markers were analyzed for all schizophrenia patients.

**Results:**

The seroprevalence of syphilis was 3.3% (52/1586) in our study. Schizophrenia patients infected with syphilis (SCZ-S) showed a higher irritability score compared with patients without syphilis (SCZ-C) (*p* < 0.05). Similarly, the serum CK, CK-MB, K and Cl values were significantly higher in the SCZ-S group than in the SCZ-C group (*P* < 0.05). In addition, the CK levels in SCZ-S drug-free patients were significantly higher than in drug-free patients in the SCZ-C group (*P* < 0.05).

**Conclusion:**

The seroprevalence of syphilis was 3.3% (52/1586) in our study, which revealed that psychotic patients were at high risk of being infected with *Treponema pallidum*. In addition, schizophrenia patients infected with syphilis can be more irritable and could have disrupted electrolyte and CK and CK-MB levels.

## Background

Schizophrenia is a complex, heterogeneous behavioral and cognitive syndrome that results from genetic and/or environmental disruption of brain development [1]. The main features of schizophrenia included positive symptoms (delusions and hallucinations), negative symptoms (impaired motivation, reduction in spontaneous speech, and social withdrawal) and cognitive impairment. However, there are no specific characteristics of schizophrenia, making it difficult for clinicians to make a precise diagnosis based on symptoms or laboratory examinations. Several articles have shown that clinical symptoms of schizophrenia, such as cognitive impairment, emotional problems, personality changes, delusions, hallucinations, and abnormal behaviors, were similar to symptoms of general paresis, which is one kind of neurosyphilis [2]. Some literature have reported that neurosyphilis patients with psychiatric symptoms were often misdiagnosed as having primary psychiatric disorders such as schizophrenia or bipolar disorder [3–5]. Previous studies showed that patients with schizophrenia may be at higher risk for acquiring syphilis [6, 7]. Several European researchers suspected bacteria to be a possible aetiological link to dementia praecox (schizophrenia) or other psychiatric disorders [6]. Whether *Treponema pallidum* could be a possible aetiological link to schizophrenia? It is still unclear whether syphilis infection could have some influence on schizophrenia or vice versa. Although some studies had focused on the characteristics of the clinical manifestations and hematological indexes in schizophrenia or neurosyphilis, few studies have reported the clinical manifestation or serum biochemical values in schizophrenia patients coinfected with syphilis. Here, we conducted a retrospective study to analyze the social function and serum biochemical values in schizophrenia patients with or without syphilis infection in Xiamen, in an attempt to find some valuable indexes for the differential diagnosis and to provide clinical care of such a special population.

## Methods

### Study population and data collection

**Fig. 1:**
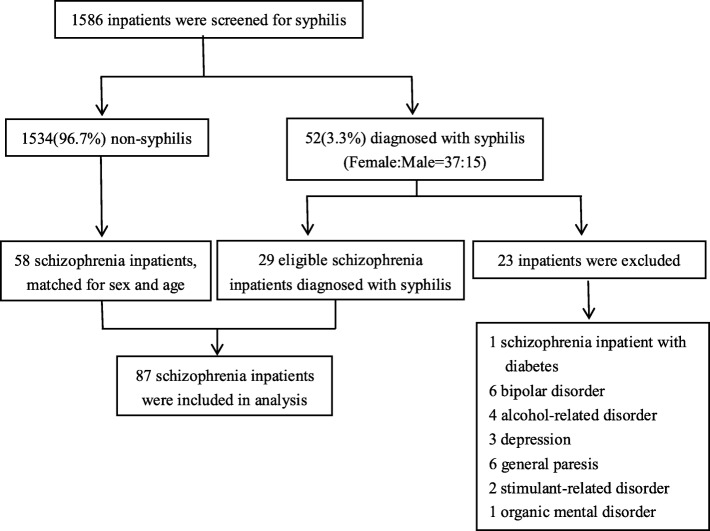
The selection criteria for this study

Patients admitted to Xiamen Xianyue Hospital, Xiamen Mental Health Center in Fujian, China, from May 2016 to August 2017 with mental illness and screened for syphilis infection were studied retrospectively. During this time, 1586 psychotic inpatients were screened for syphilis infection. A total of 87 patients were clinically diagnosed with schizophrenia by a psychiatrist according to the ICD-10, were all above 18 years old and had a record of a NOSIE-30 score. Then, 29 of these patients diagnosed with syphilis, by combining the serodiagnosis and disease history (including clinical characteristics and/or the patient’s sexual history) according to European CDCs guidelines [8], were assigned to the syphilis-positive schizophrenia (SCZ-S) group. The other patients were assigned to the control group (SCZ-C) (Fig. 1). The syphilis screening was performed using *Treponema pallidum* particle agglutination (Fujirebio, Tokyo, Japan) and automated chemiluminescence immunoassay (Roche, Mannheim, Germany). Then, syphilis patients with mental symptoms were adopt lumbar puncture for neurosyphilis diagnosis according to European CDCs guidelines. Patients with hypertension, liver disease, kidney disease, heart valve disease and diabetes mellitus and patients with human immunodeficiency virus (HIV), hepatitis B virus (HBV), hepatitis C virus (HCV).

### Sample collection and processing

Blood biochemical values and laboratory tests for syphilis were performed for all newly admitted patients within 1 day of admission. Serum biochemical tests were measured using Beckman 600 automated biochemical analyser (Beckman Coulter, Fullerton, USA) and included a liver function test (total protein (TP), alanine aminotransferase (ALT), aspartate aminotransferase (AST), AST/ALT, total bilirubin (TBIL), direct bilirubin (DBIL), indirect bilirubin (IBIL), albumin (ALB), globin (GLO), A/G, γ-glutamyltransferase (GGT), and alkaline phosphatase (ALP)), a renal function test (urea, uric acid (UA), creatinine (CREA), and glucose (GLU)), a myocardial enzymogram test (creatine kinase (CK), creatine kinase-MB (CK-MB), lactate dehydrogenase (LDH)), a blood lipid test (triglyceride (TG), cholesterol (CHO), high density lipoprotein cholesterol (HDL-C), low density lipoprotein cholesterol (LDL-C), apolipoprotein A (apoA), and apolipoprotein B (apoB)), and an electrolyte test (bicarbonate (CO_2_), phosphorus (P), magnesium (Mg), calcium (Ca), potassium (K), sodium (Na), and chloride (Cl)).

### Nurse’s observation scale for inpatient evaluation (NOSIE-30)

The NOSIE-30 [9] is a rating scale mainly used by nursing staff or other professionals in close contact with mental illness inpatients. NOSIE assesses the behaviors of inpatients diagnosed with schizophrenia across six subsets, Social Competence, Social Interest, Neatness, Irritability, Psychoticism, Retardation, by rating the frequency (never = 0 to always = 4) of 30 behaviors. The first three domains reflect positive behaviors and the last three indicate negative behaviors. NOSIE-30 was assessed within the first 3 days of admission by a trained rater in this study.

### Statistical analysis

All statistical analyses were performed using SPSS v20.0 software (SPSS Inc., Chicago, IL, USA). The results were expressed as the mean ± standard deviation. Student’s t-test (t value) and Mann-Whitney U test (Z value) were used for continuous quantitative variables with normal distributions and for variables with a skewed distribution, respectively. Chi-squared test was used to examine the potential differences in gender and treatment status. The significance level was set at *P* < 0.05.

## Results

### Demographic data of study population

From May 2016 to August 2017, 1586 psychotic patients who were screened for syphilis at Xianyue Hospital were studied retrospectively. Eighty-seven schizophrenia patients (29 SCZ-S patients and 58 SCZ-C patients) were included for further studies. The demographic characteristics of the 87 schizophrenia patients are shown in Table 1. There were no statistically significant differences in gender (χ^2^ = 0.001, *p* = 0.971) and treatment status (χ^2^ = 0.024, *P* = 0.877) between the two groups, and no differences in age (*t* = 1.928, *P* = 0.058), education (Z = − 0.732, *P* = 0.485) and mental disorder duration (Z = − 0.787, *P* = 0.436) between the groups (Table 1).

**Table 1:** Demographic characteristics of 87 schizophrenia inpatients

Variable	SCZ-S group	SCZ-C group	*P*
General			
Gender, n (%)			0.971^a^
Male	18 (62.1%)	36 (62.0%)	
Female	11 (37.9%)	22 (38.0%)	
Age (y)			0.058^b^
Mean (SD)	43.3 (11.3)	38 (11.1)	
Median (range)	44.0 (18–61)	36.5 (18–63)	
Education (y)			0.485^c^
Mean (SD)	8.64 (2.8)	9.16 (2.9)	
Median (range)	9 (6–16)	9 (6–16)	
Mental disorder duration (y)			0.436^c^
Mean (SD)	9.2 (9.9)	7.6 (7.7)	
Median (range)	7.5 (0–34)	5.0 (0–25)	
Treatment status, n			0.877^a^
Drug free	11 (37.9%)	23 (39.7%)	
Treated	18 (62.1%)	35 (60.3%)	
Psychotic phase, n			0.322^a^
Acute	8 (27.5%)	20 (34.4%)	
Recovery	11 (37.9%)	13 (22.4%)	
Chronic	10 (34.6%)	25 (43.2%)	

SCZ-S group: schizophrenia inpatients diagnosed with syphilis; SCZ-C group: schizophrenia inpatients as control

^a^*p* value calculated by Chi-squared test

^b^*P* value calculated by Student’s t-test

^c^*P* value calculated by Mann-Whitney U test

### Seroprevalence of syphilis infection in schizophrenia

In our study, 52 patients, including 37 males and 15 females (ratio 2.47:1), with different types of mental disorders were diagnosed with syphilis resulting in a prevalence of 3.3% (52/1586). There were 29 schizophrenia patients 23 patients with other types of mental disorders including six neurosyphilis (Fig. 1).

### SCZ-S patients with higher irritability scores

To assess the social function of the patients, the NOSIE-30 was conducted by a trained rater. There were no differences in social competence, social interest, neatness, psychoticism and retardation between two groups (*p* > 0.05). Interestingly, the scores of irritability in SCZ-S group were higher than the irritability scores in the SCZ-C group (Z = − 1.636, *P* = 0.004) (Table 2). The irritability scores positive correlation with CK-MB levels, while the difference was not statistically significant (*r* = 0.365, *P* = 0.052).

**Table 2:** NOSIE-30 scale between SCZ-S and SCZ-C group

	SCZ-S group	SCZ-C group	*P*
Social Competence	36.76 ± 2.80	36.67 ± 3.01	0.900
Social Interest	5.38 ± 5.00	5.18 ± 5.60	0.880^a^
Neatness	26.83 ± 4.55	26.77 ± 4.51	0.958
Irritability	9.31 ± 9.66	3.00 ± 2.60	0.004^a^
Psychoticism	1.79 ± 3.76	2.05 ± 3.83	0.782
Retardation	2.83 ± 3.23	3.54 ± 3.93	0.430^a^

Data are presented as means ± standard deviations

^a^*P* value calculated by Mann-Whitney U test

### Biochemical values in two groups

**Fig. 2:**
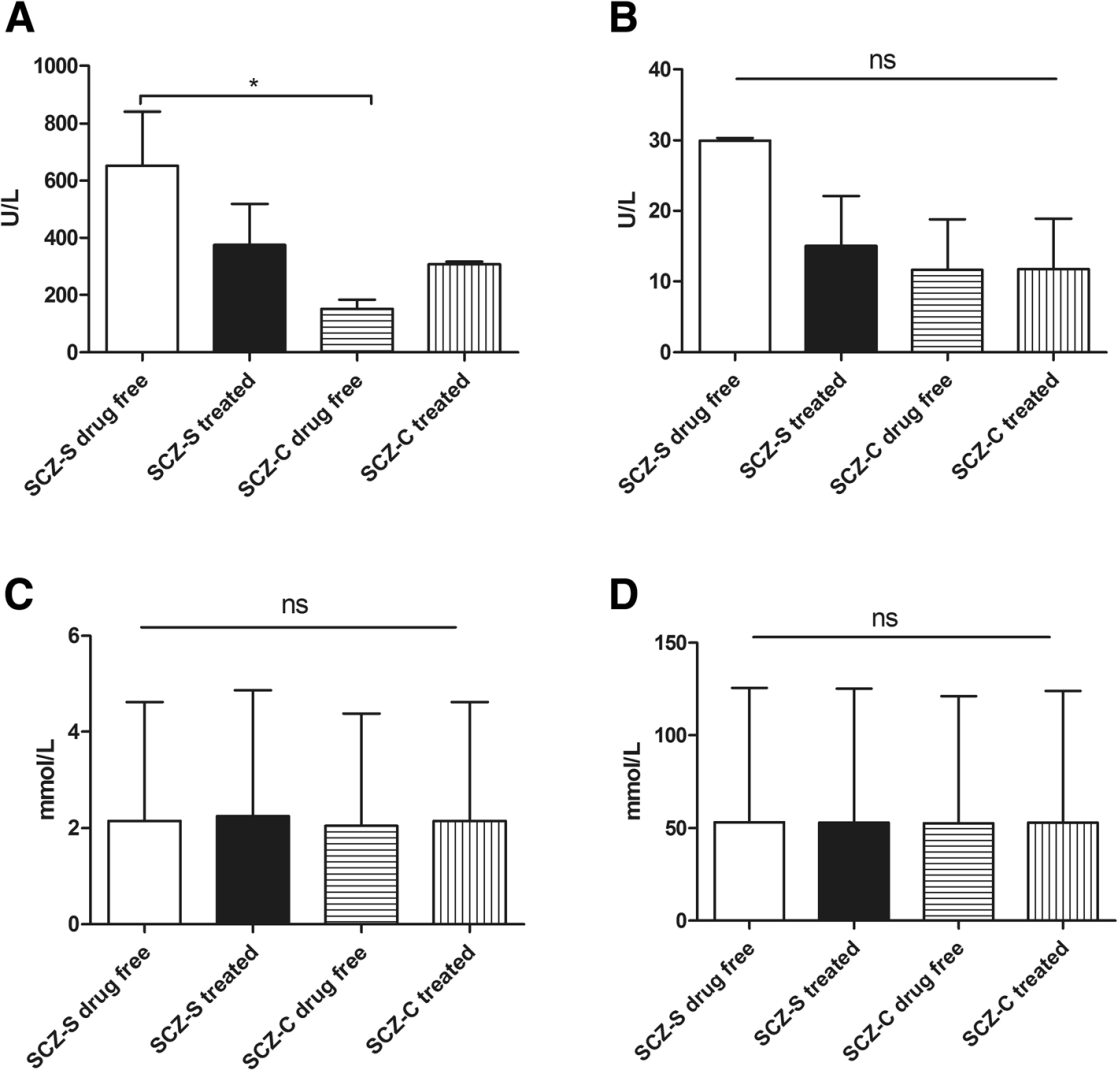
**a-d** Comparison of CK, CK-MB, K, and Cl levels between the four subgroups (SCZ-S drug-free, SCZ-S treated, SCZ-C drug-free and SCZ-C treated).*P < 0.05; ns: not significant

The serum biochemical values are shown in Table 3. Significantly higher levels of CK and CK-MB were seen in SCZ-S patients (Z = − 1.387, *P* = 0.030; *t* = 2.111, *P* = 0.039). The serum K and Cl levels in the SCZ-S group were significantly higher than these levels in the SCZ-C group (*t* = 2.207, *P* = 0.031 and *t* = 2.261, *P* = 0.011, respectively). No significant differences were found for the other biochemical values (*P* > 0.05). Furthermore, participants were divided into four subgroups according to syphilis infection and antipsychotic medication use, and there were 11 drug-free patients in the SCZ-S group and 16 in the SCZ-C group (Table 1). In the drug-free patients, the serum CK was significantly higher in the SCZ-S group than in the SCZ-C group (Z = − 1.674, *P* = 0.021) (Fig. 2).

**Table 3:** Biochemical characteristics of two groups

Blood parameters	SCZ-S group	SCZ-C group	*p*
TP (g/L)	70.59 ± 5.55	69.68 ± 6.14	0.528
ALB (g/L)	41.31 ± 4.43	42.04 ± 4.70	0.516
GLO (g/L)	29.30 ± 4.35	27.64 ± 4.00	0.106
A/G	1.44 ± 0.29	1.55 ± 0.29	0.135
TBIL (umol/L)	13.04 ± 5.59	15.09 ± 6.69	0.065
DBIL (umol/L)	2.85 ± 1.40	3.35 ± 1.47	0.164
IBIL (umol/L)	10.18 ± 4.49	12.78 ± 5.24	0.183
ALT (U/L)	30.66 ± 25.64	24.18 ± 16.45	0.206^a^
AST (U/L)	34.17 ± 33.71	25.15 ± 11.04	0.118^a^
AST/ALT	1.17 ± 0.39	1.23 ± 0.52	0.619
GGT (U/L)	24.10 ± 33.10	18.25 ± 10.92	0.300^a^
ALP (U/L)	66.24 ± 16.83	65.55 ± 14.67	0.856
TG (mmol/L)	1.24 ± 0.63	1.06 ± 1.05	0.414
CHO (mmol/L)	4.51 ± 0.99	4.81 ± 0.91	0.201
HDL-C (mmol/L)	1.28 ± 0.44	1.38 ± 0.36	0.301
LDL-C (mmol/L)	2.64 ± 0.77	1.38 ± 0.81	0.188
apoA (g/L)	1.42 ± 0.36	1.44 ± 0.27	0.812
apoB (g/L)	0.92 ± 0.24	0.93 ± 0.21	0.861
LDH (U/L)	161.97 ± 61.37	148.65 ± 43.02	0.293
CK (U/L)	360.32 ± 602.55	240.35 ± 235.28	0.030^a^
CK-MB (U/L)	23.86 ± 20.01	16.73 ± 6.53	0.039
CREA (umol/L)	78.10 ± 20.13	74.20 ± 15.33	0.364
Urea (mmol/L)	4.21 ± 3.62	3.63 ± 1.07	0.335
UA (umol/L)	355.38 ± 98.60	339.83 ± 75.31	0.460
GLU (mmol/L)	4.92 ± 0.94	5.51 ± 1.27	0.626
CO_2_ (mmol/L)	25.12 ± 1.91	25.24 ± 1.83	0.790
P (mmol/L)	1.22 ± 0.19	1.28 ± 0.24	0.311
Mg (mmol/L)	0.88 ± 0.08	0.87 ± 0.07	0.752
Ca (mmol/L)	2.27 ± 0.11	2.30 ± 0.13	0.335
K (mmol/L)	4.02 ± 0.42	3.80 ± 0.42	0.031
Na (mmol/L)	140.81 ± 2.23	139.37 ± 3.82	0.074
Cl (mmol/L)	104.23 ± 2.21	102.15 ± 3.83	0.011

Data are presented as means ± standard deviations

^a^*P* value calculated by Mann-Whitney U test

## Discussion

Previous epidemiological studies indicated that people with mental illness may be at high risk of being infected with *Treponema pallidum* [10]. However, interactions between syphilis and mental illness are poorly understood. In this study, we retrospectively studied psychotic inpatients screened for syphilis to analyze the characteristics of patients in the SCZ-C and SCZ-S groups. Our results showed that the prevalence of syphilis in psychotic inpatients was 3.3% (52/1586) which is similar to previous studies (Fig. 1) [11]. The prevalence of syphilis in this group was nearly as high as the prevalence in female sex workers (2.4–3.2%), who are regarded as a high-risk group [12]. The high prevalence of syphilis in psychotic patients may be attributed to local factors in that Xiamen is a coastal urban city in a developing country. In addition, psychotic patients have poor self-consciousness and may not be aware of the clinical manifestations of *Treponema pallidum* infection for a long time allowing the development of neurosyphilis [13, 14].

A total of 87 schizophrenia patients (29 syphilis-positive and 58 syphilis-negative patients) were included for further analysis. We evaluated the social function and serum biochemical values in this group. Social function was assessed by NOSIE-30, a scale that has been used extensively to study schizophrenia patients [15]. Our results indicated that schizophrenia patients with syphilis had a higher irritability score compared to schizophrenia patients without syphilis (*P* < 0.05). A previous study had also shown that *Treponema pallidum* infection may aggravate psychiatric symptoms [6]. Furthermore, the psychological burden of having syphilis, which is a sexually transmitted disease, may be another explanation. As clinically expected, some of the inpatients in the SCZ-S group showed high variability in mood, which became agitated more easily. This has led to suggestions that special care and restraint might be needed in this population. In addition, among the 32 kinds of serum parameters examined, we found that CK, CK-MB, K and Cl were significantly higher in the SCZ-S group than in the SCZ-C group (*P* < 0.05). It has been previously shown that serum CK levels are associated with the mood of psychotic patients [16]. Increased CK levels were found in the majority of hospitalized patients with acute schizophrenia and patients with affective psychoses [17]. Northoff and colleagues found that the serum CK levels in patients with tension-type schizophrenia was significantly higher than CK levels in healthy controls and those with non-nervous schizophrenia [18]. In addition, some authors have reported higher K and Cl levels in schizophrenia patients than in healthy controls [19–21]. Additionally, CK or K would be released into blood when the organs or tissues are damaged and the integrity of cell membrane is destroyed [22]. It has long been known that a history of *Treponema pallidum* infection can cause tissue damage and that the clinical manifestations result from the host immune response. A previous study reported that *Treponema pallidum* multiplies at the site of inoculation and immediately disseminates throughout the body, include skin [23], central nervous system [24], cardiac system, liver, spleen, and kidney [25]. As the *Treponema pallidum* continue to advance without antibiotic treatment, it can cause organ damage and can result in neurosyphilis or cardiovascular syphilis. It is known that both CK and CK-MB are biomarkers for evaluating cardiac function. When the cardiovascular system was stimulated or had an inflammation reaction, CK and CK-MB levels increase, which may explain the increased CK and CK-MB levels in syphilis-positive schizophrenia patients. Then, when *Treponema pallidum* invades the nervous system, patients could have some electrolyte imbalances, such as elevations in Cl level. Reagin, also called nontreponemal antigen, mainly comes from host tissue and cells destroyed by *Treponema pallidum*, and the intracellular K would be released at the same time. This finding is in accordance with the previous studies [17, 26].

Previous studies have associated biochemical changes with antipsychotic drug use. Nagamine et al. found that increased CK levels were more frequent in the olanzapine group [16]. Bhatia and colleagues documented a moderate degree of electrolyte imbalance in schizophrenia, and serum electrolytes became normal after treatment [21]. Therefore, we further divided these participants into four subgroups according to syphilis infection and drug taking. Elevated CK levels were found in the SCZ-S drug-free group compared to the SCZ-C drug-free group. There were no significant differences of CK-MB, K and Cl between the four subgroups (Fig. 2). We observed that the CK-MB, K and Cl levels were higher in SCZ-S drug-free patients than in SCZ-S treated patients, but there were no significant differences (*P* > 0.05). This discrepancy is probably explained by the relatively small sample size in the four subgroups. As such, a more comprehensive study is needed to demonstrate the association between syphilis and antipsychotic drug use.

The strength of our study is in its attempt to evaluate the social function and serum biochemical levels of syphilis-positive schizophrenia patients. It should be apparent from the foregoing that schizophrenic patients are at high risk of syphilis infection, and we still have much to learn about the relationship between syphilis and schizophrenia or other mental illnesses. Although there are a large number of studies have reported on syphilis, especially neurosyphilis, and mental illness, in clinical practice, it is often difficult to distinguish between a pre-existing psychiatric disorder with secondary aggravation due to neurosyphilis and a secondary psychiatric disorder as a result of neurosyphilis [6].

Of course, the shortcomings of the article must also be admitted. In the present study, social function was determined only by NOSIE. However, the NOSIE is more useful in assessing social function when it complements the Positive and Negative Syndrome Scale (PANSS) [27]. A PANSS evaluation was excluded from this study because of missing data, making it more difficult to evaluate the clinical manifestations in the schizophrenia patients. It is still unclear whether the psychiatric symptoms in neurosyphilis patients were due to nervous system injury caused by *Treponema pallidum*. This study was conducted in syphilis of any stage excluding neurosyphilis. Another limitation is that the relatively small sample size and statistical power are too low to compare biochemical levels in patient subgroups using different antipsychotic drugs.

## Conclusion

The seroprevalence of syphilis was 3.3% (52/1586) in our study, which revealed that psychotic patients were at high risk of being infected with *Treponema pallidum*. Our study showed a higher irritability score in schizophrenia patients with syphilis and higher serum CK, CK-MB, K and Cl levels. The association of psychopathology and syphilis is complex. However, the demonstrated syphilis prevalence in patients with mental illness suggests that there is a clear need for further trials to clarify the pathogenesis of syphilis in schizophrenia.

## Abbreviations

ALB: Albumin
ALP: Alkaline phosphatase
ALT: Alanine aminotransferase
apoA: apolipoprotein A
apoB: apolipoprotein B
AST: Aspartate aminotransferase
Ca: Calcium
CHO: Cholesterol
CK: Creatine Kinase
CK-MB: Creatine Kinase-MB
Cl: Chloride
CO_2_: Bicarbonate
CREA: Creatinine
DBIL: Direct bilirubin
GGT: γ-Glutamyltransferase
GLO: Globin
GLU: Glucose
HBV: Hepatitis B Virus
HCV: Hepatitis C Virus
HDL-C: High Density Lipoprotein Cholesterol
HIV: Human Immunodeficiency Virus
IBIL: Indirect bilirubin
K: Potassium
LDH: Lactate Dehydrogenase
LDL-C: Low Density Lipoprotein Cholesterol
Mg: Magnesium
Na: Sodium
NOSIE-30: Nurses’ Observation Scale for Inpatient Evaluation
P: Phosphorus
PANSS: Positive and Negative Syndrome Scale
TBIL: Total bilirubin
TG: Triglyceride
TP: Total Protein
UA: Uric Acid
